# Decoding lung-kidney interactions in sepsis: an integrated view of molecular mechanisms, pathophysiology, and therapeutic interventions

**DOI:** 10.3389/fimmu.2026.1736900

**Published:** 2026-02-18

**Authors:** Chenye Ma, Hongyi Li, Zirong Chen, Heng Song, Yuze Wang, Zhihui Liu, Xuefan Bu, Xianfei Ding, Hanfang Si, Jinghua Yang, Tongwen Sun

**Affiliations:** 1 General Intensive Care Medicine, Department of Emergency Medicine, The First Affiliated Hospital of Zhengzhou University, Henan Engineering Research Center for Critical Care Medicine, Henan Key Laboratory of Critical Care Medicine, Henan Key Laboratory of Sepsis in Health Commission, Zhengzhou Key Laboratory of Sepsis, Henan Sepsis Diagnosis and Treatment Center, Zhengzhou, China; 2 Clinical Systems Biology Key Laboratories of Henan, the First Affiliated Hospital of Zhengzhou University, Zhengzhou, Henan, China

**Keywords:** acute kidney injury (AKI), acute respiratorydistress syndrome (ARDS), lung-kidney interaction, organ crosstalk, sepsis

## Abstract

Sepsis, a great health concern globally, is characterized by multi-organ dysfunction and high mortality rate. As highly vascularized organs, the kidney and lung are the most susceptible in sepsis. Moreover, the loss of normal function in either of them may increase the risk of injury to the other, and the combined dysfunction of the respiratory system and the renal system may significantly increase the mortality rate of sepsis patients. As a part of systematic response, soluble cytokines and inflammatory mediators released from lung and kidney would exacerbate overall immune disorder, while bio-active substances like lipocalin-2, α-klotho, osteopontin released by kidney, metabolites and extracellular vesicles in sepsis can spread by circulation and induce injuries of distant tissues in lung. Furthermore, water-sodium and acid-base imbalance, as well as oxidative stress induced by kidney injury exacerbate respiratory distress in sepsis patients; while hypoxemia, hypercapnia, hemodynamic changes and endothelial injury induced by lung injury in sepsis can reduce the glomerular filtration rate. In addition, hemodynamic, neurohormonal, and immune-mediated processes induced by invasive mechanical ventilation exacerbate kidney dysfunction; pulmonary hypertension and the subsequent series of changes induced by renal replacement therapy also reduce the oxygenation in sepsis patients. As an important example of organ function network imbalance induced by sepsis, lung-kidney crosstalk involves multi-level interactions and may serve as the basis for sequential organ failure in sepsis. In this review, we summarized the scattered research advancement related to the lung-kidney interaction in sepsis, covering molecular mechanisms, pathophysiological mechanisms, as well as the impact of support therapies. Despite the lack of therapeutic targets verified by clinical research, preclinical studies have nonetheless uncovered some promising results that may offer new intervention strategies. A deep understanding of organ-organ axes represented by lung-kidney crosstalk, may provide insights into the early mechanisms of sepsis-related multiple-organ dysfunction and potential therapeutic strategies. Future research needs to distinguish the relationship between therapeutic interventions and lung-kidney interactions, integrating a broader molecular landscape and more precise animal models, or organ chips, to deeply disclose the dysregulation of organ interaction in sepsis, in order to develop more precise intervention strategies.

## Graphical Abstract

**Figure:**
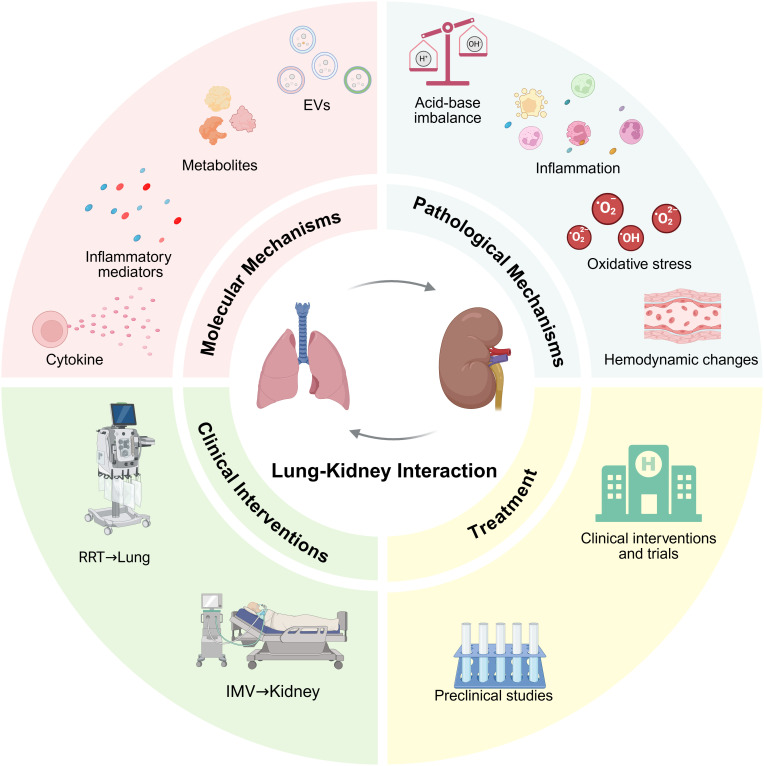

## Introduction

Sepsis is life-threatening organ dysfunction caused by a dysregulated host response to infection (1). In 2017, an estimated 48.9 million incident cases of sepsis were recorded worldwide and 11.0 million sepsis-related deaths were reported, representing 19.7% of all global deaths (2). Despite over 30 years of more than 200 randomized controlled trials, the clinical treatment for sepsis remains supportive care, antibiotic treatment and fluid resuscitation, and the mortality rates remains unacceptably high (3). The reason for this dilemma may lie in the fact that sepsis is essentially a highly heterogeneous syndrome, including pathogenesis, pathological progression, and clinical presentation, thus resulting in various patterns of organ damage (4–6), which is considered the main cause of death in sepsis (7). During the progression of sepsis, kidney and lung are particularly susceptible to damage (8). One-sixth of the inpatients in the intensive care unit (ICU) develop sepsis-associated acute kidney injury (AKI) (9); which 25 to 50% of sepsis patients experience acute lung injury (ALI) (10). More importantly, organ failure triggered by sepsis is not isolated, the dysfunction of a single organ may exacerbate the disorder of the internal environment, which may cause damage to distant organs through complex pathophysiological mechanisms, furtherly resulting in the development of multiple organ dysfunction syndrome (7, 11). For instance, the risk of AKI is doubled in patients undergoing invasive mechanical ventilation (IMV), and patients with AKI are more likely to develop acute pulmonary edema (12), and approximately 40% acute respiratory distress syndrome (ARDS) patients also develop varying degrees of AKI, with an increased mortality exceeding 50% (13). Therefore, a comprehensive and in-depth understanding of the lung-kidney interaction in sepsis is of critical importance for advancing both research and clinical management.

The mechanisms underlying lung-kidney interaction involve complex, multi-level remote crosstalk (14). In terms of molecular mechanisms, the kidneys and lungs interact through vascularly mediated soluble signaling molecules, including inflammatory factors, metabolites, extracellular vesicles (EVs) and other bio-active substances,ect (14). In terms of pathophysiological mechanisms, kidney dysfunction may lead to pulmonary edema and lung injury through water-sodium retention and disorders of substance excretion (12). As a critical organ for gas exchange, lung dysfunction can lead to decreased systemic oxygenation, which in turn affects renal perfusion and filtration functions (15). Furthermore, the lungs possess a complex immune environment, and pulmonary immune responses can further exacerbate systemic inflammation, worsening tissue damage. Kidney and lung are both organs with abundant blood flow, exhibit significant endothelial vulnerability and dysfunction in sepsis, resulting in increased vascular permeability, and hemodynamic disorders, ultimately leading to the exacerbation of organ failure (16). In terms of interventions, patients with organ dysfunction typically require supportive therapy. Renal replacement therapy (RRT) alters hemodynamics and affects pulmonary perfusion and ventilation/perfusion ratio, potentially impairing pulmonary function (17); mechanical ventilation disrupts intrathoracic pressure balance, indirectly affecting renal hemodynamics and neuroendocrine regulation (15). Given the close interaction between the lungs and the kidneys in critically ill patients, the Acute Disease Quality Initiative (ADQI) organized a highly regarded consensus conference in Innsbruck, Austria, in June 2018, and issued relevant clinical guidelines, which promoted the progress of the relevant field (18). However, in sepsis, a disease primarily characterized by organ dysfunction, research on lung-kidney interactions remains relatively limited.

In this review, we summarized the research advancement of the lung-kidney interaction in sepsis. By reviewing the current research progress, we analyze the molecular and pathophysiological mechanisms of the lung-kidney interaction in sepsis, with a focus on key molecules involved in this process. Additionally, this review also summarizes the existing evidence from clinical trials or preclinical researches, and explores how clinical interventions can address the challenges posed by lung-kidney interactions. As a typical presentation of sepsis, lung-kidney interaction may act as a trigger of multiple organ dysfunction, and relative researches may provide new insights into sepsis and offers direction for future exploration of potential therapeutic strategies.

## Molecular mechanisms

**Figure 1:**
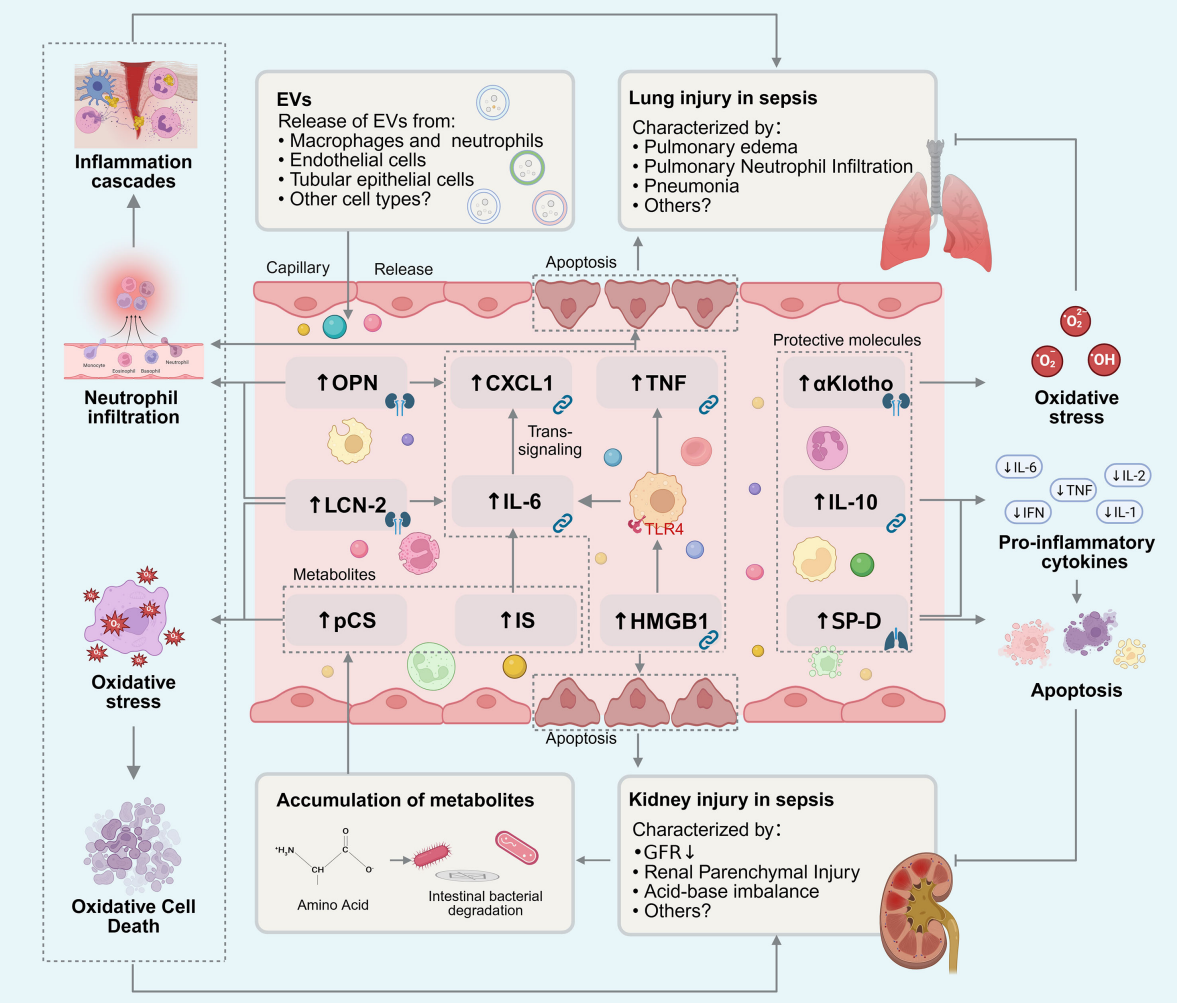
Bio-active substance-mediated lung-kidney interaction in sepsis. A variety of bio-active substances synergistically mediate signal communication and functional regulation between the kidneys and lungs in sepsis, collectively forming a complex molecular regulatory network. Pro-inflammatory and damage-associated mediators (IL-6, CXCL1, HMGB1, TNF, OPN, LCN-2) drive injury via inflammatory cascades, apoptosis, and oxidative stress. Conversely, protective molecules (αKlotho, IL-10, SP-D) mitigate tissue damage by suppressing cytokines and oxidative stress. Additionally, metabolites (IS, pCS) and EVs facilitate remote organ communication.

Organismal homeostasis relies on the coordinated interplay among specialized organs, with circulating soluble molecules serving as the primary mediators of interorgan communication network (ICN) (14). Rather than functioning in isolation, these molecules disseminate through the bloodstream to convey the molecular signals of organ injury (**Figure 1**). These diverse circulating factors, ranging from metabolites and cytokines to alarmins and exosomes, have recently been conceptualized as “ICN mediators”—key components of ICNs that regulate physiological homeostasis and propagate dysfunction to remote organs during disease (14).

### Cytokines

As central mediators of immune dysregulation, cytokines propagate and amplify inflammatory signals through the circulation. The high perfusion characteristics of the kidneys and lungs render them particularly susceptible to pro-signals. This interaction between systemic mediators and target organs not only directly induces tissue damage but also triggers a synergistic reinforcing pattern of injury through cross-transmission of inflammatory signals. In multicenter clinical trials, elevated plasma interleukin-6 (IL-6) in patients with ARDS correlate with increased subsequent risk of AKI (19). Furthermore, multiple animal studies have demonstrated that IL-6 exacerbates pulmonary neutrophil infiltration and enhances pulmonary capillary leakage after AKI; IL-6-deficient mice and anti-IL-6 antibody-treated cohorts exhibited markedly attenuated pulmonary injury (20, 21). Following infiltration of the injured kidney, leukocytes produce IL-6 when their TLR4 receptors interact with high mobility group box 1 (HMGB1) released by injured renal cells (22). Circulating IL-6 systematically enhances CXC chemokine ligand 1 (CXCL1) production and specifically promotes CXCL1-induced neutrophil infiltration in the lung possibly (21) through trans-signaling (23, 24). Furthermore, CXCL1 has been demonstrated to be associated with endothelial cell apoptosis (25), which in turn contributes to pulmonary capillary leakage (26).

Also, during AKI, the expression of genes related to the tumor necrosis factor (TNF) superfamily and apoptosis—including TNFR1, TNFR2, and TNF-α—significantly increased in rat lung microvascular endothelial cells (27, 28), accompanied by enhanced activity of Caspase3 (27). Compared to sham, the inhibition of TNF-α signaling with etanercept significantly reduces pulmonary apoptosis after AKI (27, 28); similar effects are observed in TNFR-/- mice (26, 28). These experimental findings indicate that kidney injury induces lung apoptosis predominantly via TNFR1-dependent pathways. In addition, Laura E. White et al. found that the balance between NF-κB (Complex I) and Caspase-8 (Complex II) signaling may regulate pulmonary injury and apoptosis after AKI (28).

In contrast, interleukin-10 (IL-10) inhibits the production of pro-inflammatory cytokines and chemokines (29), effectively alleviating lung inflammation induced by AKI (30). 4 hours after AKI, IL-10 knockout mice exhibited similar kidney injury and serum pro-inflammatory cytokine levels as wild-type mice; however, they displayed enhanced lung inflammation, evidenced by increased myeloperoxidase activity and elevated CXCL1 levels in the lungs (30). Furthermore, the production of IL-10 following AKI shows significant organ and cell specificity, particularly concentrated in the spleen and its immune cells, including macrophages, T cells, and B cells (30).

Notably, the relationship between the pro-inflammatory cytokine IL-6 and the anti-inflammatory cytokine IL-10 is not merely antagonistic; rather, it involves a sophisticated negative feedback regulatory mechanism. Andrés-Hernando et al. demonstrated that circulating IL-6 released following AKI serves as a critical signaling molecule, which activates the classical signaling pathway to promote phosphorylation of the transcription factor STAT3 in splenic CD4^+^ T cells, consequently specifically inducing IL-10 expression in a dose-dependent manner (30). Functioning within this negative feedback loop, IL-10 serves to constrain IL-6 production following AKI, thereby mitigating remote pulmonary inflammation (30). Also, exogenous administration of IL-10 significantly alleviates AKI-induced remote lung inflammation (31). While cytokine imbalance merely represents an epiphenomenal manifestation in septic multi-organ injury, subsequent investigations should aim to elucidate the mechanisms of upstream signaling pathways that regulate the release of key cytokines during multiple organ injury in sepsis.

### HMGB1

HMGB1, a damage-associated molecular pattern molecule, is released by necrotic cells or secreted from immune cells, triggering the inflammatory cascade. In CLP-induced sepsis models, HMGB1 interacts with tubular epithelial cells (TECs) and promote the active secretion of IL-1 and IL-6, thereby exacerbating sepsis-associated AKI (32). Doi et al. demonstrated that elevated HMGB1 induce pulmonary neutrophil infiltration and reduced vascular permeability after AKI via a TLR4-mediated pathway, whereas administration of neutralizing antibody against HMGB1 attenuated lung injury (33). It is hypothesized that HMGB1 binding to TLR4 may activate endothelial cells to express adhesion molecules, thereby triggering inflammation (34); however, this claim requires further experimental validation. Notably, HMGB1 blockade attenuated kidney ischemia reperfusion-induced lung neutrophil infiltration independent from TLR4, indicating the presence of the other HMGB1-dependent pathway that may facilitate lung inflammation after kidney ischemia reperfusion (33). For instance, HMGB1 induces cell injury via RAGE and TLR2 (33, 35). Future studies ought to thoroughly elucidate the precise molecular mechanisms by which HMGB1 mediates multi-organ injury induced by sepsis, thereby informing novel insights for the development of targeted therapeutic strategies.

### LCN-2

Lipocalin-2 (LCN-2) is an extensively expressed secretory protein that plays a pivotal role in iron sequestration during innate antimicrobial immune response. LCN-2 expression is derived from the damaged nephrons (36). A meta-analysis of biomarkers for AKI revealed that urinary and serum LCN-2 exhibits the highest diagnostic accuracy, outperforming novel biomarkers such as IL-18, L-FABP, and TIMP-2×IGFBP-7 (37). By binding to its receptor 24p3R, LCN-2 mediates iron accumulation in macrophages and instigates iron-dependent Fenton reactions that generates reactive oxygen species (ROS), and results in elevated levels of oxidative stress markers (HO-1, 4-HNE) (38); concomitantly, it facilitates the release of proinflammatory cytokines (IL-6, TNF-α) and M1 macrophage polarization, ultimately exacerbating pulmonary neutrophil infiltration and lung injury (38). Li et al. reported that LCN-2 enables early identification of sepsis-induced ARDS (AUC = 0.826) and correlates positively with neutrophil infiltration (39). Collectively, these findings indicate that LCN-2 may mediate kidney–lung crosstalk in sepsis; however, its therapeutic potential as a clinical target remains to be validated.

### SP-A and SP-D

Surfactant proteins A (SP-A) and D (SP-D) are members of the collectin subfamily within the C-type lectin superfamily (40). They constitute the primary line of defense in pulmonary innate immunity and function collaboratively to maintain alveolar immune homeostasis (40). These hydrophilic proteins recognize and bind to PAMPs on microbial surfaces through their carbohydrate recognition domains, thereby promoting pathogen clearance by phagocytes (41). Furthermore, they play a key role in fine-tuning the host inflammatory response (41). Compared with wild type mice, SP-A and SP-D double knockout mice exhibited elevated lung injury scores and a seven-fold increase in bacterial load in BALF at 24 h after being subjected to S.aureus (42). Notably, in pneumonia-induced sepsis, mice with single gene knockouts of either SP-A (43) or SP-D (44) exhibited more severe kidney damage, characterized by tubular degeneration, loss of brush border and tubular luminal cast formation when compared with wild-type mice, providing direct experimental evidence for SP-A and SP-D-mediated kidney–lung crosstalk in sepsis.

In addition, SP-A and SP-D exert synergistic protective effects, primarily through distinct but complementary mechanisms: SP-A confers cytoprotection (43), whereas SP-D functions primarily as an immune modulator (44, 45). Specifically, SP-A alleviates AKI primarily by modulating the biological activity of serum exosomes and inhibiting programmed cell death pathways, specifically apoptosis and pyroptosis, in renal tubular epithelial cells (43). Concurrently, SP-D attenuates the inflammatory cascade mediated by the NF-κB (44) and TLR4 (45) signaling axis, consequently reducing the burden of pro-inflammatory cytokines. Nonetheless, the value of SP-A and SP-D as an evaluative indicator for organ injury and prognosis in sepsis requires further investigation.

### αKlotho

αKlotho is a single-pass transmembrane protein primarily produced and secreted by the kidneys, its extracellular domain is released by secretases into blood, urine, and cerebrospinal fluid as an endocrine soluble αKlotho protein (46–48), exerting widespread pleiotropic effects on distant organs (49). As reported by Hu et al., the established model of kidney injury significantly increased serum creatinine and reduced circulating αKlotho, with oxidative damage being observed in the lungs (50). In rats, administration of αKlotho 6h after AKI alleviated lung edema, enhanced lung antioxidant capacity, and reduced lung oxidative DNA injury on day 3 after AKI (51). These experimental results suggest that αKlotho may be a protective inter-organ communication network mediator in the lung-kidney axis. Specifically, αKlotho released from the kidneys may interact with fibroblast growth factor receptor 1 on endothelial cells once in circulation, possibly initiating clathrin-mediated endocytosis and transcytosis, thereby facilitating its trans-endothelial transport to reach lung epithelial cells (52). αKlotho protects lungs through its antioxidant activity, which is partially achieved by the activation of the Nrf2 pathway (52). Nonetheless, a prospective cohort study revealed that serum αKlotho is elevated in patients with septic shock, independently associated with higher mortality (53); in patients with chronic kidney disease, serum αKlotho was elevated during sepsis and subsequently decreased as sepsis resolves (54). However, this apparent discrepancy between experimental findings and clinical observations do not negate the crucial role of αKlotho in the immune dysregulation of sepsis. Instead, the paradoxical elevation in serum levels may reflect an adaptive compensatory response to oxidative stress, or simply an increase in circulating antigenic epitopes resulting from pathological shedding. The precise mechanisms underlying this phenomenon warrant further investigation.

### OPN

Osteopontin (OPN) is an extensively distributed extracellular matrix protein that critically regulates inflammatory cell recruitment. Clinical evidence indicates that OPN increases early in sepsis and correlates with higher mortality. Elevated OPN is associated with reduced estimated glomerular filtration rate (eGFR), increased urinary albumin-to-creatinine ratio, and heightened risk of renal failure (55), positioning it as a biomarker of kidney injury (56). Through ligand-receptor pairing analysis across organs, Andreas Herrlich et al. found that OPN expressed by tubular cells engages CD44 on lung immune cells, precipitating endothelial barrier dysfunction, inflammation, and immune-cell infiltration, thereby provoking lung injury after AKI in wild-type mice (57); conversely, OPN knockout mice exhibit no such pathological manifestations (57). Additionally, OPN also promotes pulmonary neutrophil infiltration by activating the FAK-ERK/p38 signaling pathway, exacerbating ALI induced by LPS; its specific neutralization attenuates the inflammatory cascade and tissue injury (58). Nevertheless, as a pivotal mediator of innate immunity, OPN enhances macrophage phagocytosis and facilitates bacterial clearance. For instance, during Klebsiella pneumoniae-induced pneumoniae, OPN promotes host defense by enhancing the early recruitment of neutrophils to the bronchoalveolar space (59). Conversely, in patients with AKI requiring RRT, elevated baseline OPN levels have been correlated with renal functional recovery and improved survival outcomes (60). These findings reflect the differential regulatory roles of OPN in tissue injury repair under physiological versus pathological conditions. Given the structural diversity of OPN (61, 62), further research is required to determine whether these functional discrepancies stem from specific protein isoforms or post-translational modifications.

### Metabolites

In multiple organ dysfunction induced by sepsis, the kidneys and lungs form an intricate lung-kidney regulatory network orchestrated by specific metabolites; the aberrant accumulation of protein-bound uremic toxins (indoxyl sulfate [IS] and p-cresyl sulfate [pCS]) and asymmetric dimethyl-arginine (ADMA) critically amplifies inter-organ injury. Clinical studies have demonstrated that serum IS and pCS are significantly elevated in sepsis patients with AKI (63). IS can induce the expression of IL-6 in vascular endothelial and smooth muscle cells through the OAT3-mediated uptake and activation of AhR/NF-κB pathway (64), providing a mechanistic basis for its increased levels after kidney injury in sepsis and its role in promoting distal lung inflammation. For instance, research by Hideyuki Saito et al. found that IS hastened ALI following AKI by inducing AQP-5 dysfunction through the activation of p38 mitogen-activated protein kinase (MAPK) and c-Jun N-terminal kinase (JNK) signaling pathways (65). In contrast, pCS impairs the alveolar–capillary barrier by inducing intracellular ROS, activating downstream prostaglandin pathways, triggering cell death, and recruiting leukocytes to release extracellular ROS and multiplex chemoattractants (66); these effects are partially reversed by the antioxidant N-acetylcysteine (66). Furthermore, in a rat model of AKI, elevated plasma ADMA were accompanied by increased pulmonary p38 MAPK expression and enhanced heat-shock protein 27 (HSP27) phosphorylation (67). These changes were closely associated with alveolar inflammation and structural damage, suggesting a potential role of the ADMA-p38 MAPK/HSP27 axis in lung-kidney crosstalk.

### EVs

EVs are membrane-bound structures devoid of a cell nucleus, which facilitate crucial intercellular communication by transferring diverse biological cargoes, such as proteins, messenger ribonucleic acids (mRNAs), and microRNAs, between different cell types. Although the specific molecular mechanisms by which EVs mediate intercellular communication remain unclear nowadays, the crucial role of EVs in inter-organ interaction has been widely acknowledged (68, 69).

Nowadays, numerous studies have indicated that EVs serve as critical intercellular messengers in sepsis-mediated multi-organ dysfunction. For instance, in ALI induced by sepsis, macrophage-derived exosomal aminopeptidase N aggravates lung injury by regulating necroptosis of lung epithelial cells (70). Also, Qiu et al. reveal that EVs released by endothelial cells promote the reverse transendothelial migration of neutrophils through the enrichment of karyopherin subunit beta-1, thereby aggravating distant lung injury (71). In addition, the activated neutrophil (PMN)-derived exosomes are enriched with PMN elastase and perform active proteolysis unimpeded by antiproteases, leading to damage and cell apoptosis of extracellular matrix, ultimately aggravating lung injury (72). Furthermore, PMN-derived exosomes, which are rich in matrix metalloproteinase 9, contribute to the cleavage of desmoglein-2, a crucial protein for intercellular adhesion, and subsequent degradation at cell junctions, resulting in extensive tissue damage and the disruption of tissue structure (72).

Moreover, in sepsis-induced renal dysfunction, TEC-derived exosomes mediate intercellular signaling with renal macrophages, resulting in renal injury. For example, TEC-derived exosomes carrying miR-19b-3p promote M1 macrophage polarization, thereby triggering renal inflammation (73). Furthermore, TEC-derived exosomes containing C-C motif chemokine ligand 2 can activate macrophages and induce AKI (74). Similarly, exosomes released from TECs under hypoxic conditions, enriched with miR-23a, have been found to induce M1 macrophage polarization and tubulointerstitial inflammation by promoting local inflammatory responses (75).Although the role of EVs in sepsis-associated lung-kidney interaction has not been fully elucidated, it seems biologically plausible that EVs are involved in the bidirectional transport of harmful mediators between the kidneys and the lungs via endocrine action (76).

It should be noted that EVs not only participate in the transmission of harmful mediators, but also are involved in the protective mechanisms of tissue repair, as observed in models of trauma and hemorrhagic shock (77). Future research on EVs in sepsis should not only focus on their pathogenic mechanisms but also emphasize exploring the mechanisms by which they improve the disease, to identify effective strategies for reducing mortality and improving prognosis in sepsis patients.

### Others

Moreover, some molecules have been found to potentially correlate with both lung and kidney injuries in sepsis and may serve as biomarkers for sepsis-induced organ dysfunction. Through activating the ERK1/2 signaling pathway, elevated insulin-like growth factor-binding protein 7 (IGFBP-7) in septic patients can not only aggravate lung injury (78) but also regulate epithelial-mesenchymal transition to mediate kidney injury (79). Uric acid, primarily eliminated through metabolic processes and renal excretion, is known to directly impair renal function in cases of hyperuricemia. In patients with ARDS, those with high uric acid levels (≥ 3 mg/dL) demonstrated a higher mortality rate from sepsis compared to those with low uric acid levels (< 3.0 mg/dL) (80). Similarly, the kidneys are involved not only in the excretion of phenylalanine metabolites but also in the metabolic conversion of phenylalanine to maintain homeostasis (81), while phenylalanine levels were higher in the non-survivor group compared to the survivor group in ARDS patients (82). Additionally, Galectin-3 has the capability to activate platelets through its interaction with platelet GPVI, subsequently promoting macrophage polarization towards the M1 phenotype, thereby aggravating AKI (83). Galectin-3 has also been found to correlate with both APACHE II scores and the oxygenation index in ARDS patients (84). Furthermore, soluble Receptor for Advanced Glycation End-products is linked to increased 90-day mortality in ARDS patients (85) and is considered a potential therapeutic target for chronic kidney disease treatment and renal function improvement (86). AnxA1 significantly attenuates sepsis-induced lung injury by activating the FPR2-dependent endothelial nitric oxide synthase (eNOS) pathway (87); simultaneously, it also alleviates sepsis-induced kidney injury through inhibiting PI3K/AKT phosphorylation and subsequently down-regulates downstream NF-κB activity (88). Future research should thoroughly explore the specific mechanisms and potential significance of these substances in lung-kidney crosstalk induced by sepsis.

## Pathophysiological mechanisms

The pathophysiological mechanisms of lung-kidney interaction in sepsis are intricate and complex. In fact, these mechanisms share intrinsic similarities between AKI-mediated lung injury and ALI-induced kidney injury; furthermore, this bidirectional relationship is complicated by clinical interventions, such as IMV and RRT.

### Mechanisms of AKI mediated lung injury

#### Inflammatory pathophysiological mechanisms

During AKI, in addition to bio-active substances, activated immune cells are released from damaged or necrotic renal regions, triggering systemic inflammatory response syndrome, and ultimately mediating lung injury through sustained pro-inflammatory signaling cascades (89). In an adenine-induced AKI mouse model, flow cytometry showed a significant increase in CD11b^+^Ly6G^+^ neutrophils in lung tissue (90). This process is further orchestrated by CD169^+^ macrophages, which promote emergency granulopoiesis and lung neutrophil recruitment via G-CSF secretion, thereby exacerbating lung injury (90). Additionally, neutrophils and monocytes are rapidly recruited to the kidneys in the initial stages of AKI. These cells release lysosomal enzymes, resulting in TEC damage and subsequent oxidative stress, characterized by a marked increase in ROS levels in local tissues (91). The ROS-mediated MAPK and nuclear factor κB (NF-κB) signaling pathways enhance the adhesion and aggregation of inflammatory cells, thus initiating inflammatory responses both in the kidneys and in distant organs (91). Oxidative stress can induce TECs damage, and apoptotic or necrotic cells may release damage-associated molecular patterns, including heat shock proteins, histones, and HMGB1, into the extracellular space, thereby worsening lung injury (91). Furthermore, adaptive immunity is involved, as evidenced by the trafficking of CD3^+^ T lymphocytes to the lung in kidney injury murine models (92).

#### Non-inflammatory pathophysiological mechanisms

Lung injury after AKI is primarily attributed to the development of pulmonary edema (12), which is intricately associated with the disruption of sodium and water transport within pulmonary epithelial cells (93). Studies have demonstrated that AKI significantly downregulates the expression of epithelial sodium channel, Na^+^-K^+^-ATPase, and AQP-5 in the lungs (93).

Pulmonary edema can be categorized into cardiogenic and non-cardiogenic (18). Cardiogenic pulmonary edema generally arises from fluid overload (94–97) and/or cardiac dysfunction, leading to increased capillary hydrostatic pulmonary pressure and transudative pulmonary edema (12). Consequently, patients with normal kidney function can improve rapidly with fluid removal through ultrafiltration or diuresis (12). In contrast, non-cardiogenic pulmonary edema is primarily triggered by inflammatory responses, characterized by elevated inflammatory markers and neutrophil accumulation, leading to endothelial and epithelial cell damage (12). When pulmonary endothelial cells are damaged, fluid leaks directly from the capillaries into the interstitium, intensifying leakage and leading to the formation of proteinaceous pulmonary edema (12). Even with fluid removal through ultrafiltration or diuresis, patients improve only minimally, even at all (12). Importantly, many patients may fall in the spectrum between cardiogenic and non-cardiogenic pulmonary edema, suggesting a bidirectional relationship, wherein one form able to trigger or worsen the other (12).Furthermore, disruptions in fluid, electrolyte, and acid-base balance following AKI may also contribute to lung injury.

#### Crosstalk between inflammatory and non-inflammatory mechanisms

Crucially, the inflammatory and non-inflammatory mechanisms of AKI mediated lung injury are not isolated entities; rather, they engage in extensive crosstalk and form positive feedback loops that worsen pulmonary injury. Circulating pro-inflammatory cytokines have been demonstrated to directly impair alveolar fluid clearance by downregulating the expression and activity of epithelial sodium channel and Na^+^-K^+^-ATPase in alveolar epithelial cells, thereby effectively converting a systemic inflammatory insult into a functional defect in fluid transport (98). ROS trigger endothelial pyroptosis, parthanatos, and ferroptosis, compromising barrier integrity and precipitating pulmonary edema (99). Conversely, under the pathological conditions of pulmonary edema, excessive fluid accumulation and alveolar distension activate mechanosensitive ion channels, specifically Piezo1 and TRPV4, on pulmonary endothelial and epithelial cells (100). This induces calcium influx and initiates downstream inflammatory associated signaling pathways, such as the RhoA/ROCK1 pathway (100). Consequently, this cascade promotes the release of substantial amounts of pro-inflammatory cytokines and chemokines, thereby exacerbating the pulmonary inflammatory response (100).

#### Potential Impact of RRT

In ICUs, RRT is routinely implemented to mitigate the complications of sepsis. However, research pertaining to the direct effects of RRT on pulmonary function are limited, primarily derived from patients with end-stage renal disease receiving intermittent hemodialysis (IHD). In patients undergoing dialysis, the development of pulmonary hypertension is a multi-factorial, multi-mechanism pathophysiological process, encompassing pulmonary artery calcification induced by secondary hyperparathyroidism (101), vasoconstriction due to hormonal and metabolic changes (102), chronic inflammatory state (103), arterio-venous fistula formation (104, 105), volume overload (106), and hypoxemia resulting from chronic anemia (106), which may ultimately lead to pulmonary edema. Also, alternating episodes of volume depletion and overload, as well as leukocyte activation resulting from IHD, have been identified as triggers for pulmonary function deterioration (18). Moreover, rapid correction of acid-base balance during IHD could also affect the lungs (18).

These potential deleterious effects of RRT on pulmonary physiology may contribute to the adverse prognosis observed in the acute clinical setting. A multinational cross-sectional study demonstrates that critically ill patients with AKI severe enough to require RRT have worse clinical outcomes (107). The optimal timing for initiating RRT in critically ill patients has been extensively studied. However, current evidence is insufficient to support an early RRT initiation strategy compared with a delayed RRT approach for ICU patients generally and ARDS patients specifically (108, 109). Additionally, some studies have compared the impact of different RRT modalities on respiratory function in patients with AKI, having found no significant differences between the modalities (peritoneal dialysis vs. IHD (110), and IHD vs. sustained low-efficiency hemodialysis (111)). Future research should pivot from a general timing approach to identifying specific biological sub-phenotypes of AKI-ARDS patients who may selectively benefit from early RRT intervention. Also, investigations should focus on optimizing “lung-protective” RRT strategies, such as determining precise ultrafiltration rates and utilizing novel adsorptive membranes.

### Mechanisms of ALI induced kidney injury

#### Hypoxemia, hypercapnia, and hemodynamic mechanisms

**Figure 2:**
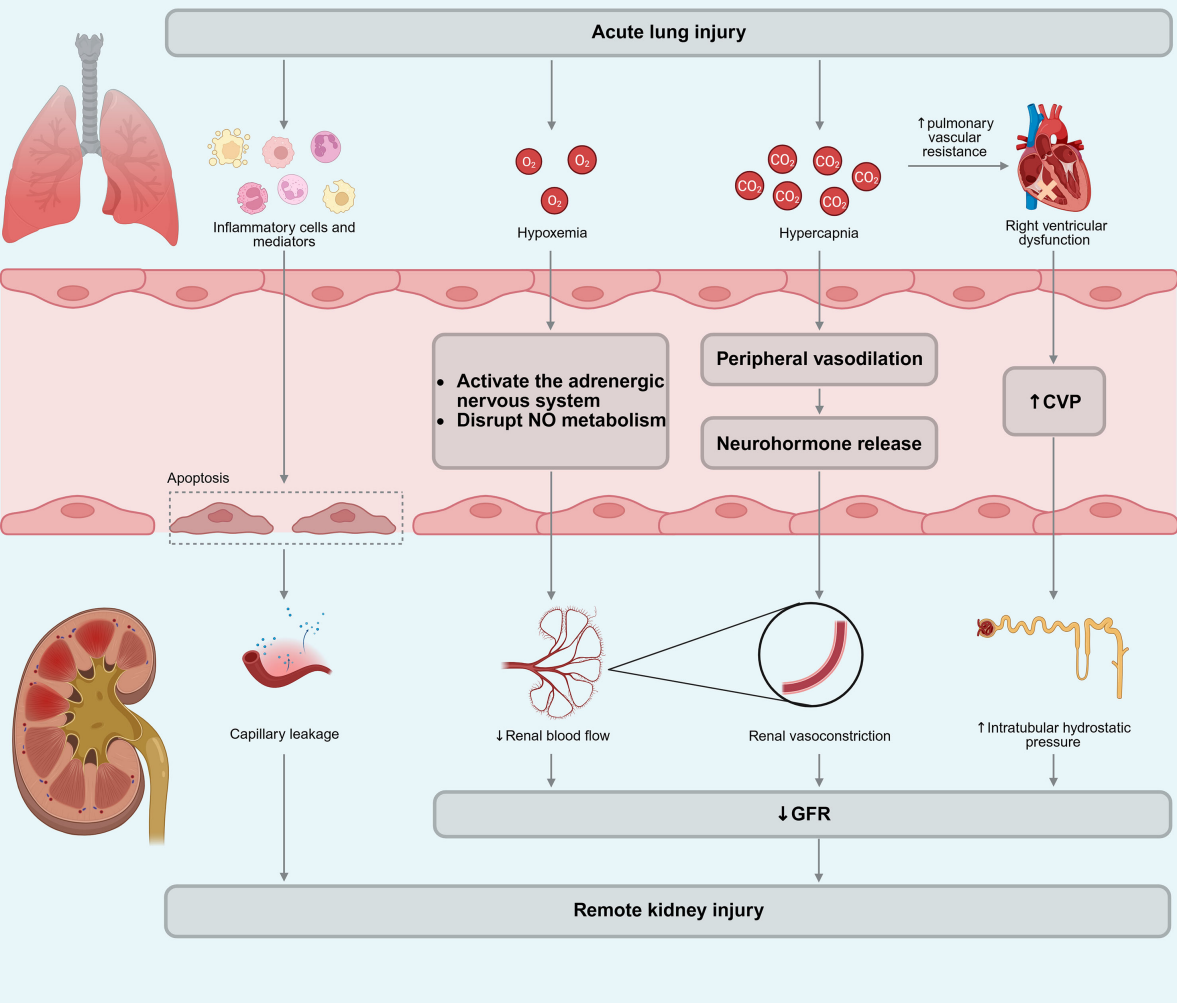
Pathophysiological mechanisms of ALI induced kidney injury. The process involves three converging pathways: inflammatory mediators causing endothelial apoptosis and capillary leakage; hypoxemia and hypercapnia inducing renal vasoconstriction and reduced blood flow; and right ventricular dysfunction leading to elevated central venous pressure. Collectively, these factors result in a decreased GFR.

The pathophysiological mechanisms of AKI in patients with ALI in sepsis involve a variety of factors, including inflammation and immune responses, hypoxemia and hypercapnia, increased central venous pressure (CVP), endothelial damage and enhanced vascular permeability (**Figure 2**) (18). Similar to lung injury caused by AKI, the inflammatory cells released from the injured lung in patients with ALI play a crucial role in the development of AKI (112). Moreover, hypoxemia diminishes RBF in a dose-dependent manner (113, 114) by various mechanisms, including stimulation of adrenergic nerves and disturbances in nitric oxide (NO) metabolism (115). Hypercapnia can directly cause renal vasoconstriction (116) or indirectly induce it through the release of neurohormones triggeered by peripheral vasodilation (114), thus altering renal hemodynamics and manifesting as a decline in GFR. Concurrently, acute hypercapnia markedly increases pulmonary vascular resistance, potentially leading to right ventricular dysfunction (117). Right heart failure, which results in increased CVP, may elevate intratubular hydrostatic pressure, thereby leading to a decline in GFR and ultimately causing AKI (118, 119). Elevated intrathoracic pressure or volume overload can also cause increased CVP, subsequently leading to renal dysfunction. Additionally, endothelial injury and increased capillary permeability constitute significant factors in renal dysfunction following ALI (16). Studies have shown that in sepsis, increased heparin-binding protein may adversely affect the kidneys by increasing endothelial permeability (120).

#### Potential Impact of IMV

**Figure 3:**
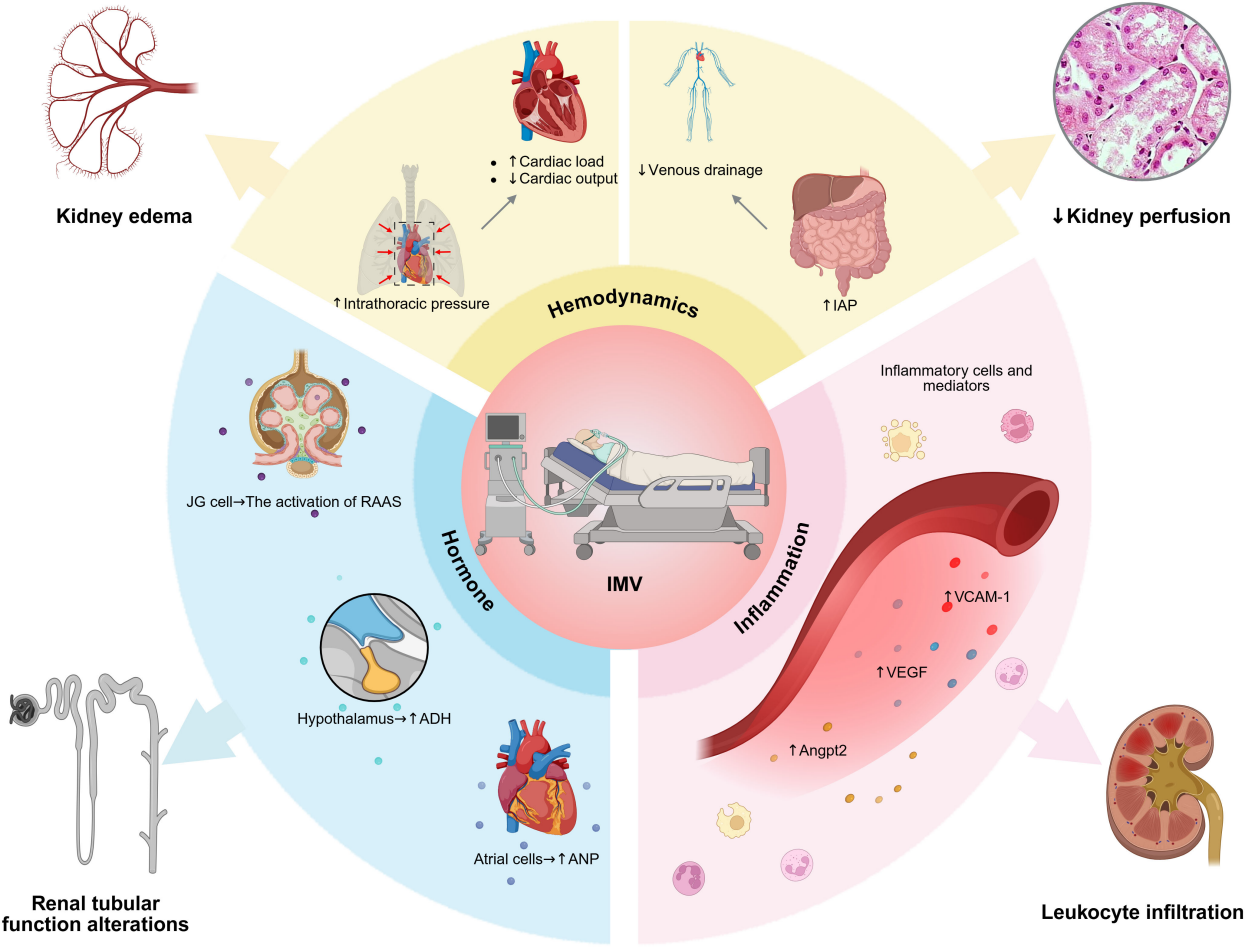
Impact of IMV on kidney function. The impact of invasive mechanical ventilation (IMV) is mediated through three pathways: hemodynamics, hormones, and inflammation. Hemodynamically, increased intrathoracic and intra-abdominal pressures reduce cardiac output and venous drainage. Hormonally, the RAAS is activated alongside elevated ADH and ANP levels. Inflammation involves the release of mediators such as VCAM-1, VEGF, and Angpt2. Collectively, these alterations lead to kidney edema, hypoperfusion, leukocyte infiltration, and tubular dysfunction.

It is widely acknowledged that the majority of sepsis patients require IMV to improve gas exchange. However, paradoxically, substantial evidence suggests that IMV frequently exacerbates kidney dysfunction (121). Given the critical role of the kidneys in maintaining homeostasis, it is crucial to elucidate the specific mechanisms by which IMV influences the development and progression of AKI. A comprehensive understanding of these mechanisms is essential not only for improving patient prognosis but also for guiding the development of targeted therapeutic strategies. The mechanisms by which IMV contributes to AKI are multi-factorial and related to incremental effects of hemodynamic, neurohormonal and immune-mediated processes (**Figure 3**) (18).

In septic lung injury, vigorous spontaneous breathing generates excessive negative pleural pressure and occult pendelluft, disrupting cardiorespiratory hemodynamics via right ventricular overload (122), and ultimately impairing renal perfusion. However, the clinical application of PEEP exacerbates hemodynamic instability, thereby imposing an additional burden on renal perfusion. Studies indicate that positive pressure ventilation and positive end-expiratory pressure (PEEP) are strongly associated with reductions in RBF, GFR, sodium excretion, and urine output (123–125). Positive pressure ventilation and PEEP can elevate intrathoracic pressure, leading to a series of dynamic physiological effects. However, excessive tidal volume may further raise intrathoracic pressure. On the one hand, elevated intrathoracic pressure can compress the mediastinal structures and pulmonary vasculature, potentially increasing right ventricular afterload and consequently reducing cardiac output and renal perfusion (126). On the other hand, increased intrathoracic pressure may impede right ventricular function (127) and decrease venous return, leading to renal congestion, as evidenced by increased CVP. Additionally, IMV may increase intraabdominal pressure (IAP) and compromise microvascular blood flow (128). Similar to systemic congestion, increased IAP may induce renal edema due to diminished venous drainage, potentially resulting in a vicious cycle that further exacerbates IAP (15).

In addition to hemodynamic alterations, neurohormonal dysregulation constitutes a critical pathway linking pulmonary dysfunction and mechanical ventilation to renal injury. The activation of the RAAS triggered by refractory hypoxemia and increased sympathetic discharge, results in severe renal vasoconstriction and ischemia (15). In addition, IMV has been demonstrated to affect the regulation of various neurohormonal systems. A study indicates that the absolute or relative intravascular volume depletion associated with IMV leads to decreased atrial stretch, which stimulates the secretion of antidiuretic hormone, ultimately resulting in fluid retention (129). Another potential mechanism for the decreased renal function and fluid retention in ventilated patients is increased renin activity mediated by the sympathetic nervous system, which decreases RBF and stimulates aldosterone, leading to decreased GFR (124, 130–132). Additionally, due to decreased venous return and reduced right atrial pressure, plasma atrial natriuretic peptide are inversely related to airway pressures during IMV (133).

In addition to hemodynamic and neurohormonal alterations, inflammatory responses also play a critical role in kidney injury during IMV. Mark Hepokoski et al. found that compared to sham and mice with sepsis from cecal ligation and puncture (CLP), mice subjected to injurious mechanical ventilation with high tidal volumes (VILI) exhibited significantly elevated vascular endothelial growth factor (VEGF) and vascular cell adhesion molecule-1 (VCAM-1) in the kidneys (134). Furthermore, compared to CLP, mice exposed to CLP followed by VILI (CLP+VILI) showed a significant increase in angiopoietin-2 (Angpt2), accompanied by higher levels of AKI biomarker (134). These findings suggest that IMV alters the expression of VEGF, VCAM-1, and Angpt2 in the kidneys, and these proteins warrant further investigation as potential biomarkers and therapeutic targets.

#### Renoprotective strategies in IMV

In the ARDSNet study, patients receiving low tidal volume ventilation exhibited lower mortality rates (135), longer time off mechanical ventilation (135), and a lower incidence of AKI (135, 136) compared to those receiving traditional mechanical ventilation. The experimental data from V. Marco Ranieri support the hypothesis that traditional mechanical ventilation may lead to an elevation in serum levels of inflammatory mediators, which are significantly correlated with the AKI (137). Therefore, monitoring tidal volumes and ventilation pressures, along with the application of lung protective ventilation strategies, is recommended for patients receiving IMV to reduce the risk of new or worsening AKI (18). Notably, compared to gradual reductions, abrupt decreases in PEEP in ARDS rats led to more severe lung injury and significantly increased expressions of IL-6 and VEGF (138). Therefore, gradual adjustments in ventilation settings are crucial.

In the FACTT-trial patients with ARF/ARDS receiving IMV showed improved lung function after the implementation of a conservative fluid strategy, with a trend toward reduced use of RRT (139). The FACTT Lite trial confirmed that fluid restriction not only significantly increased ventilator-free days but also notably reduced AKI rates (140). Additionally, a meta-analysis showed that inhaled nitric oxide (iNO), used as an adjunctive treatment for refractory hypoxemia and pulmonary hypertension, may increase the risk of kidney injury in ARDS patients (141). Consequently, the use of specific ancillary interventions known to be associated with AKI is advised against, including liberal fluid administration, nephrotoxin exposure and high doses of iNO (18).

IMV may induce significant alterations in renal hemodynamics through elevations in intrathoracic and IAP. Hence, meticulous monitoring and timely intervention in factors predisposing to renal dysfunction in patients undergoing mechanical ventilation, such as hypotension, venous congestion, right ventricular failure and d intraabdominal hypertension, are critical.

## Treatment

Currently, definitive interventions for the prevention and treatment of lung-kidney interaction in sepsis remain elusive. However, preclinical studies targeting sepsis-related organ dysfunction are providing new insights for treatment. Additionally, drugs developed based on known mechanisms of lung-kidney crosstalk are being investigated for their potential to positively modulate this interaction, though clinical efficacy is yet to be fully established.

### Preclinical studies

Although there is currently no single approach that can definitively prove its significant efficacy in mitigating the lung-kidney interaction caused by sepsis, many preclinical studies have been progressively initiated based on existing molecular and pathophysiological insights (**Table 1**). Nevertheless, the clinical applicability of these findings remains constrained, as standard animal models often oversimplify the variable onset time, pathogen load, and standard-of-care interventions inherent to human sepsis.

**Table 1:** Preclinical studies on the treatment of sepsis induced renal and pulmonary injury.

Authors (Year)	Study subjects	Intervention	Outcome	Lessons learned	Ref.
Zhao et al. (2021)	C57BL/6J mice, renal IR model	Sodium hydrosulfide administered intraperitoneally	Improved lung histological injury scores and the wet/dry weight ratio, increased PaO_2_/FiO_2_, reduced inflammatory cells and cytokines in BALF	Sodium hydrosulfide protects against renal IR induced lung injury, mitochondrial dysfunction, and inflammation via Nrf2-mediated NLRP3 pathway inhibition.	(142)
Nishida et al. (2020)	Male C57BL/6N mice, renal IR model	Human serum albumin-thioredoxin fusion protein administered by intravenous injection immediately and 24 h after renal IR	Attenuated renal IR injury and associated lung injury, suppressed oxidative stress and inflammation in kidney and lung, inhibited neutrophil infiltration and apoptosis in the lung	Human serum albumin-thioredoxin fusion protein has a therapeutic potential in preventing AKI and AKI-associated lung injury through its systemic and sustained multiple biological actions.	(143)
Hayase et al. (2020)	Male C57BL/6J mice, renal IR model	Pretreatment (30 min before renal IR) and delayed treatment (6 hours after renal IR) with recombinant thrombomodulin	Pretreatment and delayed treatment: decreased pulmonary MPO activity, proinflammatory cytokines, vascular leakage, and lung damage; no effect on renal dysfunction	Recombinant thrombomodulin protects lungs after renal IR by blocking histone and NET accumulation in lungs.	(144)
Du et al. (2020)	Male C57BL/6J mice, renal IR model	GSK484, a peptidyl arginine deiminase-4 inhibitor, administered daily by intraperitoneal injection for 3 days before the operation	Reduced pulmonary pathological changes, neutrophil infiltration, NET formation, apoptosis, and inflammatory factor secretion	GSK484 has anti-inflammatory and antiapoptotic effects against ALI induced by renal IR injury.	(145)
Rossi et al. (2019)	C57BL/6 wild-type mice, renal IR model	Hemin administered intraperitoneally 24 hours before surgery	Improved renal outcomes after renal IR reduced systemic inflammation and lung inflammation	Hemin-induced heme oxygenase-1 mitigates AKI and AKI-induced ALI by reducing inflammation and mitigating oxidative damage.	(146)
Liu et al. (2018)	Male Sprague-Dawley rats, renal IR-mediated ALI model	Artesunate intraperitoneal injected 1hour before renal IR treatment	Attenuated lung damage, vascular permeability, edema, and inflammation; reduced inflammatory cells and cytokines in BALF; inhibited NLRP3 inflammasome activation	Artesunate pretreatment attenuated renal IR-mediated ALI through reducing ROS-induced activation of the NLRP3 inflammasome.	(147)
Azarkish et al. (2013)	Male Wistar rats, renal IR model	N-acetylcysteine treated before IR and daily after IR for next 3 days	Reduced serum creatinine and BUN, attenuated the lung injury score	Low dose of N-acetylcysteine may protect the kidney function and lung tissue damage after kidney IR.	(148)
Si et al. (2013)	Male Sprague-Dawley rats, LPS model	3-aminobenzamide, a poly (adenosine diphosphate-ribose) polymerase inhibitor, administered after intratracheally instilled with LPS	Improved pulmonary edema and oxygenation, preserved renal function	The poly (adenosine diphosphate-ribose) polymerase inhibition attenuated lung-kidney crosstalk induced by intratracheal LPS instillation, partly via an inhibition of NF-κB dependent proinflammatory cytokines.	(149)
Bhargava et al. (2013)	Adult male C57B/6 mice, LPS or CLP model	TNF-α antibody administered intraperitoneally 2 hours before or after LPS	Prophylactic TNF-α antibody: reduced serum cytokines, lung MPO activity, BUN; Post-sepsis administration: no effect	ALI and AKI are improved with pre, but not post, sepsis administration of TNF-α antibodies.	(150)
Awad et al. (2011)	Adult male Sprague-Dawley rats, renal IR model	Curcumin administered orally for 5 days before operation	Decreased systemic and blood levels of cytokines, reduced lung injury	Curcumin protects against renal IR injury and distant organ injury via immune-mediated and anti-apoptotic mechanisms.	(151)

IR, ischemia-reperfusion; BALF, bronchoalveolar lavage fluid; Nrf2, nuclear factor erythroid 2 - related factor 2; NLRP3, nucleotide-binding oligomerization domain-like receptor protein 3; AKI, acute kidney injury; MPO, myeloperoxidase; NET, neutrophil extracellular trap; ALI, acute lung injury; ROS, reactive oxygen species; ICAM1, intercellular cell adhesion molecule-1; TNF-α, tumor necrosis factor-α; BUN, blood urea nitrogen; LPS, lipopolysaccharide; CLP, cecal ligation and puncture.

#### Preclinical studies to mitigate lung injury in AKI

As previously mentioned, inflammatory cells and cytokines play a pivotal role in organ dysfunction induced by sepsis. Recently, a substantial number of animal studies have been conducted around the regulation of inflammatory mediators. For instance, recombinant thrombomodulin (144) and peptidyl arginine deiminase-4 inhibitor GSK484 (145), by mitigating neutrophil infiltration and neutrophil extracellular traps formation, alleviate lung injury induced by renal ischemia-reperfusion. Curcumin manifests protective effects on lung injury induced by renal ischemia-reperfusion through the attenuation of inflammatory cytokines production and inhibition of pulmonary tissue apoptosis pathways (151). α-melanocyte-stimulating hormone protects against both kidney and lung injury after renal ischemia by inhibiting the activation of transcription factors and stress genes (152).

Furthermore, the inhibition of oxidative stress has also garnered significant attention. In preclinical trials, a variety of antioxidants exhibited positive treatment prospects in preventing and treating lung-kidney interaction in sepsis. For instance, human serum albumin-thioredoxin fusion protein (143) and N-acetylcysteine (148), through the inhibition of inflammatory responses and attenuation of oxidative stress, have been shown to reduce alveolar epithelial cell damage and impairment of alveolar-capillary barrier function, thereby effectively attenuating kidney injury and its associated lung injury. Sodium hydrosulfide (142) and artesunate (147), by curtailing oxidative stress and reducing the activation of the NLRP3 inflammasome, exhibit protective effects on distal lung mediated by kidney injury. The induction of heme oxygenase-1 has been demonstrated to provide a protective effect on kidney injury, which can be achieved through hemin pretreatment; however, its role in mitigating ALI induced by AKI remains to be extensively investigated (146). These preclinical studies offer invaluable experimental evidence for a profound understanding of the pathophysiological mechanisms underlying AKI induced lung injury and for the identification of novel therapeutic targets. Nevertheless, these findings warrant further validation and optimization with the aim of translating into clinical practice, thereby propelling the development of effective therapeutic strategies.

#### Experimental approaches to protect kidney function in ALI

Compared with lung injury induced by AKI, preclinical study concerning the mitigation of ALI induced kidney injury is relatively sparse. This discrepancy may be associated with the complexity of the mechanisms underlying kidney injury induced by ALI, coupled with the challenge in detecting early pathological alterations. Nevertheless, existing preclinical studies have unveiled potential mechanisms of kidney injury induced by ALI and have explored corresponding intervention strategies. For example, Chieko Mitaka et al. found that inhibiting poly (adenosine diphosphate-ribose) polymerase could attenuate lung-kidney crosstalk induced by LPS, partly through an inhibition of NF-κB dependent proinflammatory cytokines (149). Additionally, Sarah Faubel et al. found that administering TNF-α antibodies before, rather than after, sepsis can improve AKI or ALI in mice (150). Given the current scarcity of preclinical studies addressing kidney injury induced by ALI, future research should place further emphasis on the molecular mechanisms and pathophysiological processes of kidney injury in ALI, alongside a systematic evaluation of potential intervention targets and therapeutic strategies.

#### Therapeutic potential of EVs in the lung-kidney interaction in sepsis

Currently, the therapeutic potential of EVs in organ dysfunction and inter-organ crosstalk induced by sepsis has attracted significant attention. A study has confirmed that mesenchymal stem cell-derived EVs can induce the transition of AMs from the pro-inflammatory M1 phenotype to the anti-inflammatory M2 phenotype through mitochondrial transfer (153). Specifically, specific EV-shuttled miRNA (miR146a) plays a crucial role in this regulatory process (153). Notably, there exists similar reparative mechanisms based on M2 macrophages in both the lung and kidney, which provides a theoretical basis for the development of EV-based therapies for multi-organ damage (154). In addition, EV-mediated crosstalk between TECs and neighboring macrophages, as well as between pulmonary epithelial cells and AMs, constitutes a key mechanism of damage in AKI and ALI (76, 155). EVs derived from mesenchymal stromal cells with immunomodulatory and tissue-repair functions, are expected to treat AKI and ALI by inhibiting abnormal crosstalk between the aforementioned cells (68). Furthermore, EVs derived from epithelial or endothelial cells can protect endothelial cells, reduce the increase in cell permeability induced by LPS, thereby alleviating inflammatory damage and providing a new etiological therapeutic strategy for related diseases (156).

However, EV-based therapeutic strategies still face numerous challenges, such as the preparation of EVs, quality control, and the evaluation of safety and efficacy in clinical applications. Therefore, future research should focus on the key aspects that could facilitate the implementation of these therapies and their translation from basic research to clinical practice: the possibility of EVs manipulation to enrich them with drugs or protective miRNAs to target specific cell types (for example, ECs) of lung or kidney (157); standardized methods for EVs isolation and storage; the selection of EVs administration routes [for example, inhalation (158, 159)]; the availability of new biomarkers to assess the efficacy of MSC-derived EVs after administration (160, 161).

### Clinical interventions and trials

Research into preventive and therapeutic strategies for lung-kidney interaction in sepsis, and their clinical implementation, continues to encounter challenges. Currently, in addition to the recommendations by Kidney Disease: Improving Global Outcomes (KDIGO), no proven specific interventions exist to prevent or treat the lung-kidney interaction in sepsis. The consensus report of the ADQI 21 Workgroup provides some recommendations for clinical practice, including conservative fluid management, selected use of diuretics or ultrafiltration, lung-protective ventilation, and early recognition and treatment of lung infections (18).

As a critical mechanism of lung-kidney interaction in sepsis, targeting inflammatory mediators can effectively mitigate lung or kidney injury induced by sepsis (**Table 2**). Based on this, modulating inflammatory mediators may offer a promising therapeutic approach for addressing lung-kidney interaction in sepsis and improving patient prognosis. Intravenous administration of CYT107, a glycosylated recombinant human IL-7, has been shown to significantly extend organ support free days in patients with septic shock complicated by severe lymphopenia (163). Similarly, the neutrophil elastase inhibitor Sivelestat has been demonstrated to significantly increase ventilator-free days and improve the 180-day survival rate in patients with ALI associated with SIRS (177). Furthermore, recombinant human IL-11 significantly reduced APACHE II scores and 28-day mortality in in sepsis patients accompanied with thrombocytopenia by attenuating inflammatory responses and facilitating platelet recovery (178). In addition, Mycobacterium w, an immunomodulator, has demonstrated the potential in improving the prognosis of sepsis patients, including reducing mortality, shortening mechanical ventilation time and ICU length of stay, and decreasing the incidence of secondary bacterial infections (175). However, its direct impact on organ function and the underlying mechanisms require further investigation (173). Although anti-TNF-α fragment antibody AZD9773 (176), TREM-1 inhibitor Nangibotide (164, 174), non-neutralizing adrenomedullin antibody adrecizumab (165, 179), and the anti-inflammatory agent prolonged-release pirfenidone (171) have all shown good tolerance and safety in the treatment of sepsis-related diseases, they have not demonstrated significant efficacy in improving key clinical outcomes, such as renal function, SOFA score, and 28-day mortality. This highlights the need for further research to optimize therapeutic strategies or explore more effective interventions.

**Table 2:** Clinical trials on the treatment of sepsis induced organ dysfunction: Interventions with defined mechanism.

Authors (Year)	Study design	Patients	Intervention	Outcome	Lessons learned	Ref.
Gottlieb et al. (2024)	RCT	209 patients hospitalized with COVID-19 and severe or critical disease	Standard of care plus sirukumab vs. standard of care plus placebo	Time to sustained clinical improvement up to day 28: no difference (*p* = 0.849); mortality at day 28: no difference (*p* = 0.806)	In critical COVID-19 patients who received sirukumab, there was no statistically significant difference in time to sustained clinical improvement versus placebo.	(162)
Daix et al. (2023)	RCT	21 patients with septic shock and lymphopenia	CYT107, a glycosylated recombinant human IL-7 vs. placebo	Organ support free days: significant increase (*p* < 0.05); 7-day CD4^+^ and CD8^+^ T cell counts: significant increase (*p<*0.005)	CYT107 treatment effectively reversed sepsis-induced lymphopenia and reduced the duration of organ support.	(163)
François et al. (2023)	RCT	335 non-COVID-19 patients with septic shock	Three group: 1.low dose nangibotide 2.high dose nangibotide 3.placebo	The mean difference in SOFA score from baseline to day 5: no difference (Group1 vs. Group3) (*p* = 0.80), no difference (Group2 vs. Group3) (*p* = 0.104)	Nangibotide did not significantly improve SOFA score at the predefined sTREM-1 value.	(164)
van Lier et al. (2022)	RCT	301 adults in the early phase of septic shock	Adrecizumab (HAM 8101) vs. placebo	Change from baseline SOFA score after 24 h: significant reduction (*p* = 0.045); 28-day mortality: no difference (*p* = 0.094)	Adrecizumab did not significantly improve organ function in patients with septic shock.	(165)
Sivapalasingam et al. (2022)	RCT	1822 patients with critical COVID-19 receiving mechanical ventilation	Sarilumab vs. placebo	The proportion of patients with ≥1-point improvement in clinical status from baseline to day 22: no difference (*p* = 0.3261)	Sarilumab did not show efficacy in hospitalized patients with severe/critical COVID-19.	(166)
Lonze et al. (2022)	RCT	178 adult patients with severe COVID-19 disease and hyperinflammation	High-dose clazakizumab vs. placebo	28-day ventilator-free survival: significant increase (OR = 3.84; p [OR > 1] = 99.9%); odds of intubation: significant reduction (OR = 0.2; p [OR] < 1; 99.9%)	Clazakizumab improved 28-day ventilator-free survival, clinical outcomes in patients with COVID-19 and hyperinflammation.	(167)
RECOVERY Collaborative Group (2021)	RCT	4116 adult patients with COVID-19	Usual standard of care alone vs. usual standard of care plus tocilizumab	28-day mortality: significant reduction (*p* = 0.0028); receipt of invasive mechanical ventilation: significant reduction (*p* = 0.0019)	Tocilizumab improved survival and other clinical outcomes in hospitalized COVID-19 patients with hypoxia and systemic inflammation.	(168)
Salama et al. (2021)	RCT	389 patients hospitalized with COVID-19 pneumonia not receiving mechanical ventilation	Standard care plus tocilizumab vs. standard care plus placebo	Mechanical ventilation or death by day 28: significant reduction (*p* = 0.04)	In hospitalized patients with COVID-19 pneumonia who were not receiving mechanical ventilation, tocilizumab reduced the likelihood of progression to mechanical ventilation or death, but it did not improve survival.	(169)
Rosas et al. (2021)	RCT	452 patients hospitalized with severe COVID-19 pneumonia	Tocilizumab vs. placebo	Clinical status at day 28 (ordinal scale): no difference (*p* = 0.31); mortality at day 28: no difference (*p* = 0.94)	The use of tocilizumab did not result in better clinical status or lower mortality than placebo at 28 days in patients with severe COVID-19 pneumonia.	(170)
Chávez-Iñiguez et al. (2021)	RCT	88 septic acute kidney injury patients	Three group: 1.PR-PFD at 1,200 mg/day 2.PR-PFD at 600 mg/day 3.placebo	The serum creatinine reversion rate: no difference (*p* = 0.70); urinary volume: no difference (*p* = 0.47); mortality: no difference (*p* = 0.38)	PR-PFD did not improve the clinical course of septic acute kidney injury and seemed to be safe in terms of adverse events.	(171)
Lescure et al. (2021)	RCT	420 adults with COVID-19 requiring oxygen supplementation or intensive care	Three group: 1.sarilumab 400 mg 2.sarilumab 200 mg 3.placebo	Median time to an improvement of two or more points: no difference (Group1 vs. Group3) (*p* = 0.34), no difference (Group2 vs. Group3) (*p* = 0.96)	Sarilumab did not show efficacy in patients with COVID-19 and receiving supplemental oxygen.	(172)
Sehgal et al. (2021)	RCT	202 patients with severe sepsis	Mw vs. placebo	28-day mortality: significant reduction (*p* = 0.04); ventilator-free days: no difference (*p* = 0.82); delta SOFA score: no difference (*p* = 0.18)	The use of Mw was associated with a significant reduction in mortality in patients with severe presumed gram-negative sepsis.	(173)
François et al. (2020)	RCT	49 patients with septic shock	Nangibotide vs. placebo	SOFA score LS mean change from baseline to day 5: no difference (*p* = 0.56)	No significant increases in treatment emergent adverse events were detected in nangibotide-treated patients.	(174)
Sehgal et al. (2015)	RCT	50 adult patients with severe sepsis	Mw vs. saline	The days on mechanical ventilator: significant reduction (*p* = 0.025); the delta SOFA score: significant reduction (*p* = 0.027)	The use of Mw in severe sepsis was associated with significant reduction in days on mechanical ventilation, and lower delta SOFA score.	(175)
Bernard et al. (2014)	RCT	296 adult patients with severe sepsis and/or septic shock	Three group: 1.low dose AZD9773 2.high dose AZD9773 3.placebo	Mean number of ventilator-free days: no difference (Group1 vs. Group3) (*p* = 0.36), no difference (Group2 vs. Group3) (*p* = 0.51)	AZD9773 rapidly and efficiently decreased plasma TNF-α concentration in patients with severe sepsis/septic shock, but this effect did not translate into clinical benefit.	(176)
Aikawa et al. (2011)	Prospective Cohort Study	221 patients with ALI associated with SIRS	Sivelestat vs. control	The adjusted mean number of ventilator-free days: significant increase (*p* = 0.0022); the adjusted 28-day ventilator-weaning rate: significant increase (*p* = 0.0028); the adjusted 180-day survival rate: significant increase (*p* = 0.0022)	Sivelestat contributed to early weaning from the mechanical ventilation, while showing no negative effect on the long-term outcomes of ALI associated with SIRS.	(177)
Wan et al. (2015)	Case-Control Study	105 patients with severe sepsis and thrombocytopenia	IL-11 therapy vs. conventional therapy	28-day mortality rate: significant reduction (*p* = 0.037); APACHE II score from days 3 to 14: significant reduction (*p* < 0.05)	IL-11 has a protective role and can reduce the mortality in sepsis patients accompanied with thrombocytopenia.	(178)

RCT, Randomized Controlled Trial; COVID-19, coronavirus disease 2019; SOFA, sequential organ failure assessment; TREM-1, triggering receptor expressed on myeloid cells-1; PR-PFD, prolonged-release pirfenidone; Mw, Mycobacterium w; IL, interleukin; APACHE, II acute physiology and chronic health evaluation II; TNF-α, tumor necrosis factor-α; ALI, acute lung injury; SIRS, systemic inflammatory response syndrome.

Concurrently, immunomodulatory interventions targeting COVID-19-associated sepsis offer additional insights into therapeutic strategies for lung-kidney crosstalk in sepsis. Among these agents, therapies targeting the IL-6 pathway are the most extensively studied. Clazakizumab, a direct IL-6 inhibitor, has been shown to significantly prolong 28-day ventilator-free survival in hospitalized patients with COVID-19 and hyperinflammation and improve related clinical outcomes (167). However, Sirukumab, another IL-6 inhibitor, has not demonstrated similar efficacy in clinical trials (162). The study conducted by the RECOVERY Collaborative Group indicated that Tocilizumab, a monoclonal antibody against the IL-6 receptor, can improve survival and related clinical outcomes in hospitalized COVID-19 patients with hypoxia and systemic inflammation (168). In contrast, studies by Salama and Rosas et al. reached opposing conclusions (169, 170), highlighting the need for further exploration of the potential of Tocilizumab in sepsis treatment. Studies by Lescure et al. and Sivapalasingam et al. failed to confirm the efficacy of Sarilumab, anthoer IL-6 receptor antibody, in critically ill COVID-19 hospitalized patients, suggesting that more data is needed to support its clinical application (166, 172). While studies on COVID-19 provide valuable insights into immune-targeted therapies for lung-kidney interactions in sepsis, substantial heterogeneity exists between viral and bacterial sepsis in terms of PAMPs, the immunological characteristics of cytokine storms, and the underlying mechanisms of host immune paralysis. Consequently, future translational research is imperatively needed to delineate differential immunomodulatory strategies tailored to specific pathogen-induced sepsis subtypes.

Additionally, drugs proven effective against sepsis-induced kidney injury or lung injury likely improve lung-kidney interaction by modulating their complex interaction mechanisms during sepsis, offering new therapeutic insights and strategies for multi-organ dysfunction in sepsis (**Table 3**). The continuous statin therapy also benefits patients with severe sepsis- associated ARDS (190). Janz et al. revealed that administering acetaminophen within 24 hours of ICU admission may reduce oxidative injury and improve renal function for adult patients with severe sepsis and detectable plasma cell-free hemoglobin (189). Moreover, Xuebijing (182), Coenzyme Q10 (183), and ulinastatin (181) have been shown to improve certain clinical parameters in patients with sepsis. In addition, the combination therapy of vitamin C and thiamine, with or without hydrocortisone was associated with significant reduction in SOFA score among patients with sepsis and septic shock, although it had no impact on short-term mortality (180). These discoveries not only augment our understanding of the lung-kidney interaction in sepsis but also proffer a broader spectrum of therapeutic options to clinicians, potentially enhancing patient outcomes and life quality.

**Table 3:** Other clinical trials on the treatments of sepsis induced organ dysfunction.

Authors (Year)	Study design	Patients	Intervention	Outcome	Lessons learned	Ref.
Yao et al. (2021)	Meta-analysis of RCTs	1,428 patients with sepsis and septic shock	Combination therapy of thiamine, vitamin C, and hydrocortisone vs. placebo	Delta SOFA score: significant reduction* (*p* < 0.001); duration of vasopressors usage: significant reduction* (*p* < 0.001)	The combination therapy was associated with significant reduction in organ dysfunction.	(180)
Wang et al. (2019)	Meta-analysis of RCTs	1,358 patients with sepsis, severe sepsis, or septic shock	Ulinastatin vs. control	All-cause mortality: 22.42% vs. 34.65% (*p* < 0.00001); the incidence of MODS: 16.28% vs. 39.21% (*p* < 0.00001)	Ulinastatin may be an effective treatment for sepsis and septic shock, improving all-cause mortality and other related outcomes.	(181)
Liu et al. (2023)	RCT	1,817 patients with sepsis	Xuebijing vs. placebo	28-day mortality rate: 18.8% vs 26.1% (*p* < 0.001); 6-day delta SOFA score: -1.7 vs.-2.4 (*p* < 0.001)	The administration of Xuebijing reduced 28-day mortality and improved SOFA score at day 6 among patients with sepsis.	(182)
Soltani et al. (2020)	RCT	40 septic patients	Coenzyme Q10 vs. control	7-day SOFA score: 6.5 vs. 7.2 (*p* = 0.55); 7-day TNF-α: 35.3 vs. 47.3 (*p* = 0.003); 7-day MDA: 12.4 vs. 42.8 (*p* = 0.003); in-hospital mortality: 20% vs. 65.5% (*p* = 0.01)	Coenzyme Q10 has a positive effect on clinical parameters as well as mitochondrial dysfunction when administered in the early phase of sepsis, but it does not improve organ function.	(183)
Douglas et al. (2020)	RCT	124 patients with septic shock	Personalized resuscitation strategy based on change in stroke volume after passive leg raise vs. usual care	Required RRT: 5.1% vs. 17.5% (*p* = 0.04); required mechanical ventilation: 17.7% vs. 34.1% (*p* = 0.04)	A personalized resuscitation strategy is safe and may result in lower mortality and faster resolution of organ dysfunction.	(184)
Zampieri et al. (2020); Hernández et al. (2019)	RCT	424 patients with septic shock	Peripheral perfusion-targeted resuscitation vs. lactate-targeted resuscitation	28-day mortality: 34.9% vs. 43.4% (*p* = 0.06); the SOFA score: significant reduction* (*p* = 0.02)	Peripheral perfusion–targeted resuscitation may result in lower mortality and faster resolution of organ dysfunction compared with a lactate-targeted resuscitation strategy.	(185, 186)
Brown et al. (2019); Semler et al. (2018)	RCT	15,802 critically ill adults; 1,641 critically ill adults with sepsis	Balanced crystalloids vs. saline	30-day in-hospital mortality: 26.3% vs. 31.2% (*p* = 0.01); major adverse kidney event within 30 days: 14.3% vs. 15.4% (*p* = 0.04)	Balanced crystalloids were associated with a lower rate of major adverse kidney events, including death, new RRT, or persistent renal dysfunction compared to saline.	(187, 188)
Janz et al. (2015)	RCT	245 patients with severe sepsis and detectable cell-free hemoglobin	Acetaminophen vs. placebo	2-day F_2_-Isoprostanes: 24 pg/mL vs. 36 pg/mL (*p* = 0.047); 3-day serum creatinine: 1.04 mg/dL vs. 1.36 mg/dL (*p* = 0.039)	Acetaminophen may reduce oxidative injury and improve renal function in severe sepsis patients with detectable plasma cell-free hemoglobin.	(189)
Mansur et al. (2015)	Prospective Observational Cohort Study	404 patients with sepsis-associated ARDS	Statin therapy vs. No statin therapy	28-day survival: 88.5% vs. 62.5% (*p* = 0.0193); the SOFA score: 8.9 vs. 9.9 (*p* = 0.0158)	Statin therapy has a beneficial effect on 28-day survival and organ failure in patients with severe sepsis-associated ARDS.	(190)
Yunos et al. (2012)	before-after study	1,533 critically ill adults	Chloride-restrictive intravenous fluid administration strategy vs. chloride-liberal intravenous fluid administration strategy	Incidence of AKI: 8.4% vs 14% (*p* < 0.001); use of RRT: 6.3% vs. 10% (*p* = 0.005)	The implementation of a chloride-restrictive strategy was associated with a significant decrease in the incidence of AKI and use of RRT.	(191)

*Specific value not mentioned.

RCT, Randomized Controlled Trial; COVID-19, coronavirus disease 2019; ARDS, acute respiratory distress syndrome; SOFA, sequential organ failure assessment; TNF-α, tumor necrosis factor-α; MDA, malondialdehyde; RRT, renal replacement therapy; MODS, multiple organ dysfunction syndrome; AKI, acute kidney injury.

In clinical practice, the management of sepsis and septic shock focuses on infection control, fluid management, and hemodynamic stabilization. Although these measures are fundamentally designed to improve systemic survival, clinical studies have shown that they also play a critical role in mitigating organ dysfunction, offering new perspectives for treating sepsis and its inter-organ interaction. A plethora of cohort studies have indicated that delayed administration of antibiotic may significantly increase the incidence of AKI and ALI in patients with septic shock, potentially decreasing their survival rate (192, 193). Subsequently, during the procession of fluid resuscitation, the use of balanced crystalloids can reduce the incidence of major adverse kidney events within 30 days compared with the use of normal saline (187, 188). Studies by Hernández and Zampieri et al. found that peripheral perfusion-targeted resuscitation, compared to lactate-targeted resuscitation, can lower mortality rates and expedite the recuperation of organ functionality (185, 186). It’s worth noting that patients adopting a chloride-restrictive strategy reduce the risk of AKI and RRT compared to their counterparts following a chloride-liberal approach (191). Concurrently, Douglas et al. demonstrated that personalized resuscitation strategies are not only safe but may also precipitate a decline in mortality rates and expedite the restoration of organ function (184), emphasizing the importance of individualized treatment strategies in the management of sepsis.

## Conclusions and perspectives

Ultimately, through a multi-level research framework, these efforts will promote the clinical translation of lung-kidney interaction in sepsis research and drive breakthroughs in prevention and treatment strategies. However, current epidemiological studies on sepsis-induced lung-kidney crosstalk face challenges in eliminating the potential effects of clinical interventions on organ interactions. Furthermore, the understanding of potential mediators facilitating this crosstalk remains insufficient. Therefore, future studies should integrate multi-omics data to decipher the specific molecular pathways and regulatory networks underlying lung-kidney interactions. Additionally, the development of more precise models—such as organoid co-culture systems or organ-on-a-chip technology—is essential to establish a specific sepsis model that simulates isolated lung-kidney crosstalk, thereby eliminating the interference from pre-existing organ injuries and other organ dysfunction. Given that current therapeutic strategies primarily target common injury pathways rather than organ-specific crosstalk mechanisms, future interventions should leverage precision-targeting technologies, such as nanoparticle-based delivery and genetic regulation tools, to specifically block the pathological communication between the kidneys and lungs. Ultimately, a cross-disciplinary research framework is needed to accelerate the clinical translation of lung-kidney interaction in sepsis research and drive breakthroughs in prevention and treatment strategies.

## Glossary

ADMA: asymmetric dimethyl-arginine
ADQI: Acute Disease Quality Initiative
AKI: acute kidney injury
ALI: acute lung injury
Angpt2: angiopoietin-2
ARDS: acute respiratory distress syndrome
CLP: cecal ligation and puncture
CVP: central venous pressure
CXCL-1: CXC chemokine ligand 1
EVs: extracellular vesicles
GFR: glomerular filtration rate
HMGB1: high mobility group box 1
HSP27: heat-shock protein 27
IAP: intraabdominal pressure
ICN: interorgan communication network
ICU: intensive care unit
IGFBP-7: insulin-like growth factor-binding protein 7
IHD: intermittent hemodialysis
IL-6: interleukin-6
IL-10: interleukin-10
IMV: invasive mechanical ventilation
iNO: inhaled nitric oxide
IS: indoxyl sulfate
JNK: c-Jun N-terminal kinase
KDIGO: Kidney Disease: Improving Global Outcomes
LCN-2: lipocalin-2
MAPK: mitogen-activated protein kinase
mRNAs: messenger ribonucleic acids
NF-κB: nuclear factor κB
NO: nitric oxide
OPN: osteopontin
pCS: p-cresyl sulfate
PEEP: positive end-expiratory pressure
RCT: randomized controlled trial
ROS: reactive oxygen species
RRT: renal replacement therapy
SP-A: surfactant protein A
SP-D: surfactant protein D
TEC: tubular epithelial cell
TNF: tumor necrosis factor
VCAM-1: vascular cell adhesion molecule-1
VEGF: vascular endothelial growth factor.
